# Prognostic and Predictive Value of CD163+ Macrophages for Neoadjuvant Chemotherapy in Osteosarcoma

**DOI:** 10.3390/biomedicines14050991

**Published:** 2026-04-26

**Authors:** Yuejun Luo, Bo Wang, Wanjiang Feng, Zibo Xu, Hongyu Wu, Ziming Yan, Haoyu Guo, Weifeng Liu

**Affiliations:** 1 Department of Orthopaedic Oncology Surgery, Beijing Jishuitan Hospital, Capital Medical University, Beijing 100035, China; yuejunluo_daisy@163.com (Y.L.); drwangbo@pku.edu.cn (B.W.); fengwanjiang@cimrbj.ac.cn (W.F.); xzb200186@126.com (Z.X.); wuhongyu@pku.edu.cn (H.W.); loaded_yzm1216@163.com (Z.Y.); 2 National Center for Orthopedics, Beijing 100035, China

**Keywords:** osteosarcoma, biopsy, neoadjuvant chemotherapy, CD163+ macrophage, tumor microenvironment

## Abstract

**Background**: The CD163+ macrophage is considered a key component of the tumor immune microenvironment (TIME) in osteosarcoma (OS) in relation to tumor progression and chemotherapy resistance. However, the relationship between CD163+ macrophage infiltration and the efficacy of neoadjuvant chemotherapy (NACT) in OS remains unexplored. **Methods**: This study collected a total of 195 biopsy samples from newly diagnosed, pretreated OS patients. The infiltration of CD163+ macrophages was evaluated using immunohistochemical (IHC) staining with anti-CD163 antibody. Additionally, multiplex fluorescence staining (CD8, CD68, CD163, and PDL1) was employed to further characterize the TIME profiles associated with different levels of CD163+ macrophage infiltration. The relationships between various clinical characteristics, survival outcomes, and CD163+ macrophage infiltration levels were also assessed. **Results**: CD163+ macrophages in biopsy tissues ranged from 2.25 cells/mm^2^ to 3974.79 cells/mm^2^ and 1.37 cells/mm^2^ to 3027.20 cells/mm^2^ in the training and validation cohorts respectively. Multivariate analysis identified that CD163+ macrophage density was an independent predictor for NACT response. Importantly, patients with high CD163+ macrophage infiltration exhibited poorer DFS, DMFS, and RFS than their counterparts. **Conclusions**: CD163+ macrophage infiltration was an independent predictor for NACT response. Patients with high CD163+ macrophage density benefited less from NACT and exhibited a more immunosuppressive TIME than low-density patients.

## 1. Introduction

Osteosarcoma (OS) is a primary malignant bone tumor that predominantly occurs in children and adolescents, accounting for 5–10% of all pediatric malignant bone tumors [1,2]. This malignancy is characterized by extremely high aggressiveness and a strong invasive potential, often arising in the metaphysis of long bones, severely compromising limb function and quality of life in affected individuals [3,4,5]. The disease imposes a substantial burden on young patients. The current standard treatment regimen for OS primarily involves surgery combined with chemotherapy, supplemented by radiotherapy and targeted therapy [3,4]. Notably, the continuous advancements in neoadjuvant chemotherapy treatment (NACT) for OS have significantly improved patient survival rates [6,7].

However, the clinical outcomes of neoadjuvant chemotherapy in OS patients vary significantly. Currently, patients who achieve a major pathological response (MPR) after NACT tend to derive greater survival benefits from the overall treatment [8]. Moreover, patients with non-MPR are generally regarded as having a poor chemotherapy response. These non-MPR patients often experience limited therapeutic benefits from the conventional treatment regimen [9,10]. Moreover, in some cases, tumor progression during neoadjuvant chemotherapy necessitates more-extensive surgical resection upon subsequent intervention. Therefore, identifying precise biomarkers predictive of MPR in OS may assist clinicians in developing more-accurate and personalized treatment strategies for these patients.

The tumor immune microenvironment (TIME) is the complex biological milieu in which tumor cells survive and proliferate, comprising diverse immune cells, stromal cells, and extracellular components [11]. It plays a critical role in determining therapeutic responses. Among its key cellular constituents are immune cells, including tumor-associated macrophages (TAMs), myeloid-derived suppressor cells (MDSCs), and lymphocytes [12,13]. In OS, the TIME is typically characterized by a higher infiltration of myeloid-lineage immune cells and a relative paucity of lymphocytes, contributing to a profoundly immunosuppressive landscape [14,15]. Emerging evidence highlights the pivotal role of TAMs in the OS TIME, particularly their involvement in mediating chemotherapy resistance [16,17]. Studies suggest that M2 macrophages are closely associated with chemoresistance mechanisms, making them a significant factor in treatment failure [18,19,20,21]. However, there is currently no comprehensive large-cohort clinical study elucidating the relationship between macrophages and chemotherapeutic response in OS.

In this study, we collected 195 OS biopsy samples and, for the first time, employed immunohistochemistry (IHC) and multiplex immunofluorescence (mIF) to systematically investigate the infiltration of immunosuppressive macrophages in OS. Furthermore, we comprehensively analyzed its association with neoadjuvant chemotherapy response and clinical outcomes, which may provide reliable clues for precision medicine in clinical practice.

## 2. Materials and Methods

### 2.1. Patients and Tissue Samples

In this retrospective study, we analyzed 195 OS biopsy specimens obtained from patients who underwent biopsy and surgical resection at Beijing Jishuitan Hospital from January 2021 to April 2025. The study was approved by the Institutional Research Ethics Committee of Beijing Jishuitan Hospital. Written informed consent was obtained from all enrolled patients. The inclusion criteria were treatment-naïve patients with newly diagnosed OS who underwent initial biopsy followed by standardized neoadjuvant chemotherapy and surgical resection. All pathological diagnoses were independently reviewed and confirmed by two board-certified pathologists. The flow diagram describing the sample section is displayed in Figure S1.

Clinical data from all patients were independently collected and verified by two investigators. BMI was computed as weight in kilograms divided by the square of height in meters (kg/m^2^). Participants were stratified into two groups: underweight/normal weight (BMI < 25 kg/m^2^) and overweight (25–29.9 kg/m^2^)/obese (BMI ≥ 30 kg/m^2^). Recurrence-free survival (RFS) was defined as the time from the date of biopsy to the first occurrence of local recurrence or distant metastasis or the last follow-up. Disease-free survival (DFS) was identified from the biopsy time to the occurrence of any disease recurrence, metastasis, or death from any cause or the last follow-up. Distant metastasis-free survival (DMFS) was the time from biopsy-confirmed diagnosis to the first occurrence of metastasis. Overall survival (OS) was calculated from the biopsy date until death or the last follow-up. This study is reported based on the REMARK criteria [22].

### 2.2. Immunohistochemistry and Multiplex Immunofluorescence Staining

In this study, formalin-fixed, paraffin-embedded (FFPE) tumor biopsy sections (4 μm thick) were used for immunohistochemical staining. Sections were deparaffinized in absolute ethanol, followed by rehydration in phosphate-buffered saline (PBS). Antigen retrieval was performed using EDTA (pH 9.0) in 30 min. Then, sections were incubated in 3% hydrogen peroxide (H_2_O_2_) to block endogenous peroxidase activity. Non-specific binding was minimized by incubating the sections with 3% bovine serum albumin (BSA). Sections were incubated overnight at 4 °C with the appropriately diluted primary antibody (CD163, Abcam#182422, clone number EPR19518, 1:600, Cambridge, UK). After primary antibody incubation, slides were washed three times with PBS on a shaking platform. Sections were then incubated with the corresponding horseradish peroxidase (HRP)-conjugated secondary antibody for 50 min at room temperature. Immunoreactivity was visualized using 3,3′-diaminobenzidine (DAB) as the chromogen. Nuclei were counterstained with hematoxylin for approximately 3 min, followed by rinsing under running tap water. Sections were dehydrated through a graded series of ethanol, cleared in xylene, and mounted with a coverslip using a permanent mounting medium.

FFPE tissue sections (4 μm) were subjected to the same deparaffinization, antigen retrieval, and blocking procedures as described in the immunohistochemistry (IHC) protocol above. Following blocking, sections were incubated with the appropriate fluorophore-conjugated primary antibody (CD8 (Abcam#237709, clone number CAL66, 1:750), CD68 (Abcam#213363, clone number EPR20545, 1:1000), CD163 (Abcam#182422, clone number EPR19518, 1:600), and PDL1 (Abcam#205921, clone number 28-8, 1:400)), followed by the corresponding secondary antibody. Signal amplification was performed using tyramide-based fluorescence reagents (TSAs) to enhance detection sensitivity. Cell nuclei were counterstained with 4′,6-diamidino-2-phenylindole (DAPI). Fluorescence images were acquired at the respective excitation/emission wavelengths using a fluorescence microscope equipped with appropriate filter sets.

### 2.3. Digital Image Acquisition and Quantification of CD163+ Macrophages

In this study, we employed Aipathwell, an artificial intelligence-powered digital pathology image analysis software (Wuhan Servicebio Biotechnology Co., Ltd., Wuhan, China), to quantify CD163+ macrophage infiltration within tumor tissues from each patient. The CD163+ macrophage count for each case was determined by averaging the positive cell numbers across three randomly selected high-power fields (HPFs, 200× magnification). To mitigate sampling bias in heterogeneous tumors, the 3 HPFs were selected from the most invasive tumor margins (“hotspots”) by a senior pathologist blinded to clinical outcomes. Using the median value of CD163+ macrophage infiltration intensity from the training cohort as the cutoff, patients were stratified into high- and low-infiltration groups. This classification was subsequently applied to the validation cohort.

### 2.4. Statistical Analysis

For data analysis and visualization, we utilized statistical software including SPSS (version 25.0), GraphPad prism (version 10), and R (version 3.5.1). Associations between CD163+ macrophage infiltration density and clinical characteristics were evaluated using chi-square tests or Fisher’s exact tests, as appropriate. Differences in chemotherapy-induced necrosis rates among patients with varying CD163+ macrophage infiltration levels were compared using Wilcoxon rank-sum tests. Survival analysis was performed using the Kaplan–Meier method to estimate DFS, DMFS, RFS, and OS among different patient subgroups, with between-group comparisons assessed by log-rank tests. Patients who did not experience the event of interest by the study’s data cutoff date, or those who were lost to follow-up, were right-censored at the date of their last documented clinical contact. Univariate and multivariate Cox proportional hazards models were employed to identify independent predictors. In the multivariate regression analysis, clinically relevant variables were also included based on the prior literature and biological plausibility, regardless of their univariate *p*-value. Model performance was comprehensively assessed using ROC-AUC analysis, calibration plots (with Hosmer–Lemeshow goodness-of-fit testing), and decision curve analysis (DCA). Throughout this study, a two-sided *p*-value < 0.05 was considered statistically significant.

## 3. Results

### 3.1. Patient Characteristics

The information on clinicopathological features from the enrolled 195 patients in training cohort and validation cohort is displayed in the Table 1. Totally, 195 OS patients with NACT before surgical operation from Beijing Jishuitan Hospital from January 2021 to April 2025 were included. Postoperative pathological evaluation revealed an MPR rate of 46.15% (90/195) across all enrolled patients. Specifically, MPR was achieved in 45.30% (53/117) of the training cohort and in 47.44% (37/78) of the validation cohort. Baseline characteristics differed between cohorts: the training cohort had 13 upper limb and 104 lower limb cases, versus 11.11%/88.89% in the validation cohort. Median ages were 15.98 and 16.17 years, respectively. Other variables (weight, sex, hematologic profiles) are shown in Table 1. Specifically, a total of 40 DFS events, 36 DMFS events, five RFS events, and seven OS events were documented in the entire cohort. Meanwhile, the median follow-up is now reported as 20 months (95%CI: 15.0–24.0), 22 months (95%CI: 18.0–26.0), 18 months 95%CI: 15.0–22.0), and 21 months 95%CI: 18.0–24.0) in DFS, DMFS, RFS, and OS, respectively (estimated by the reverse Kaplan–Meier method).The Kaplan–Meier analyses revealed that patients with MPR exhibited a tendency toward superior DFS, DMFS, and RFS in the training, validation, and entire cohorts (Figure S2).

### 3.2. CD163+ Macrophages’ Infiltration and Clinicopathologic Features

**Figure 1:**
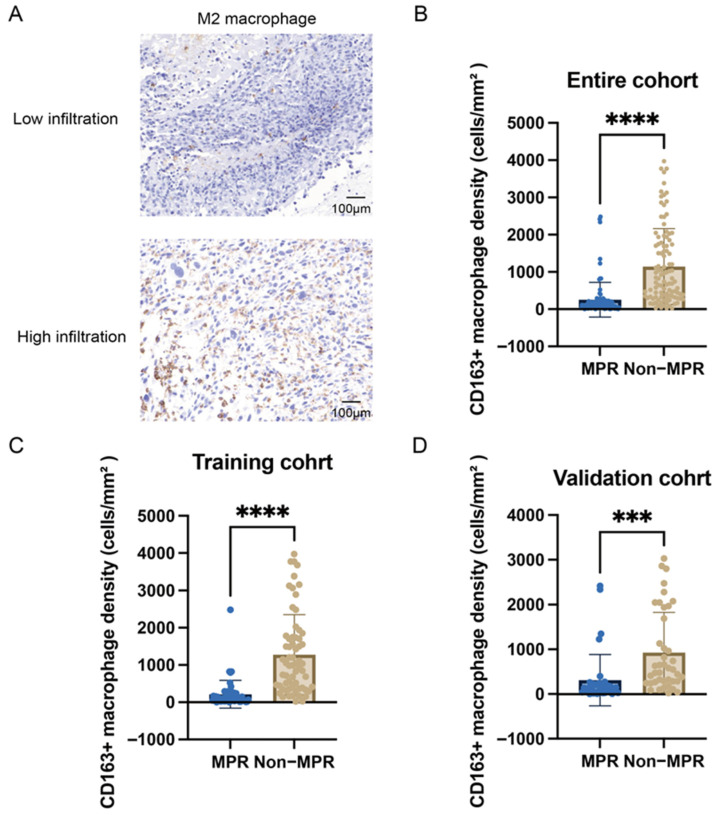
CD163+ macrophage infiltration and the association with clinicopathologic features in osteosarcoma. (**A**) The representative images of high and low CD163+ macrophage infiltration in biopsy tissues of OS. The distribution of CD163+ macrophage density in MPR and non-MPR groups in the entire (**B**), training (**C**), and validation (**D**) cohorts. *** indicates *p* < 0.01, and **** indicates *p* < 0.001.

**Figure 2:**
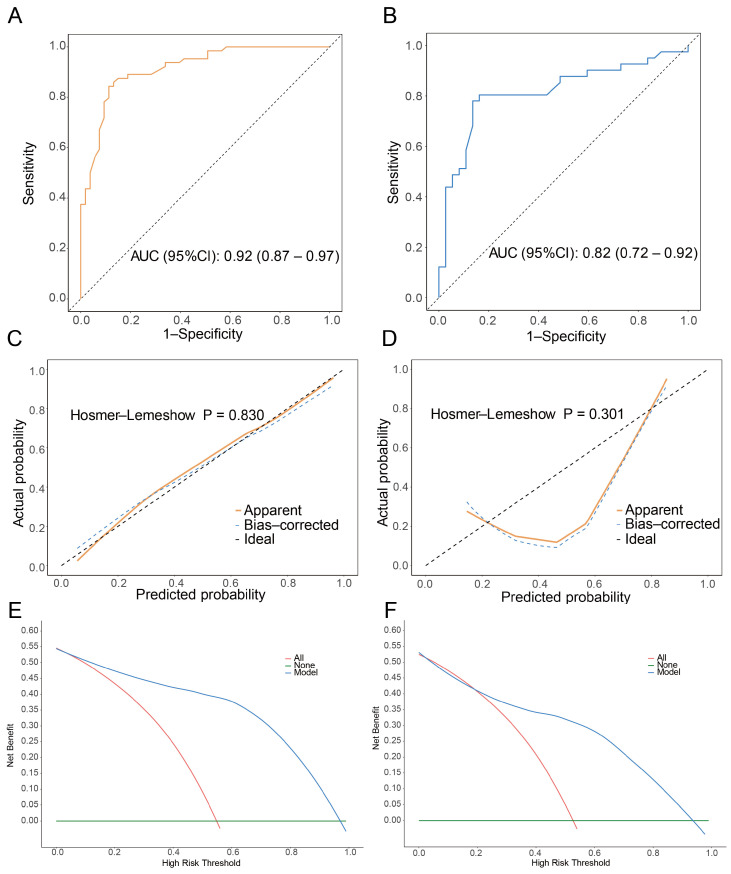
Evaluation of CD163+ macrophage infiltration in the pathological responses in osteosarcoma. (**A**,**B**) The ROC for the performance of the CD163+ macrophage density in the training and validation cohorts (The curve for the training cohort is shown in yellow, and that for the validation cohort in blue. (**C**,**D**) The Hosmer–Lemeshow test for the performance of CD163+ macrophage density in the training and validation cohorts. (**E**,**F**) The DCA analysis for the performance of CD163+ macrophage density in the training and validation cohorts.

CD163+ macrophages play a crucial role in the TIME of OS, where they are closely associated with tumor invasiveness, metastasis, and immune responses. The CD163+ macrophages’ density was evaluated in pretherapeutic biopsy samples, using the IHC method with CD163 antibody in the training and validation cohorts. The representative images are shown in Figure 1A. The median count of M2 macrophages was 334.47 cells/mm^2^, ranging from 2.25 cells/mm^2^ to 3974.79 cells/mm^2^ in the training cohort, while it was 251.11 cells/mm^2^ (1.37 cells/mm^2^ to 3027.20 cells/mm^2^) in the validation cohort. There is a statistically significant difference between pathological responses and CD163+ macrophage density. The relationship between CD163+ macrophage density in the pre-NACT biopsies and pathological response is exhibited in Figure 1B–D. Compared with NACT responders (tumor necrosis rate, TNR ≥ 90%), non-responders (TNR < 90%) demonstrated significantly higher CD163+ macrophage intensity. Then, patients were stratified into high- and low-infiltration groups based on median cutoff of CD163+ macrophage density. Importantly, the predictive value of CD163+ macrophage intensity was also investigated. The overall accuracy of CD163+ macrophage intensity was 86% (95%CI: 0.79–0.92), with a receiver operating characteristic (ROC) curve of 0.92 (95%CI: 0.87–0.97) in the training cohort, while it was 0.82 (95%CI: 0.79–0.92) in the validation cohort (Figure 2A,B). The full ROC metrics are also shown in Table S1. Meanwhile, the Hosmer–Lemeshow test indicated good calibration in both the training (*p* = 0.830) and validation (*p* = 0.301) sets, with predicted probabilities closely matching observed outcomes, as reflected by the calibration curve aligning well with the ideal diagonal (Figure 2C,D). Furthermore, we also performed decision curve analysis (DCA) to evaluate the clinical net benefit of the predictive model more comprehensively. The results showed that, in both the training and validation sets, the model curve lay above the treat-all and treat-none lines, indicating that using this model for decision-making offers greater clinical value than blind intervention or no intervention (Figure 2E,F).

In addition, we evaluated the predictive value of CD163+ macrophage infiltration density, both as a continuous variable and after logarithmic transformation, for the response to neoadjuvant chemotherapy. The results showed that, whether as a continuous variable or following logarithmic transformation, it effectively predicted the necrosis rate of neoadjuvant chemotherapy. The corresponding results are presented in Table S2. Given that the immune microenvironment may influence the response to TKIs differently than cytotoxic chemotherapy, we performed sensitivity analyses in both the overall cohort and the cohort excluding patients treated with TKI agents. The results indicated that CD163+ macrophage infiltration retained favorable predictive value for the pathological necrosis rate after neoadjuvant chemotherapy in both settings (Tables S3 and S4).

### 3.3. Prognostic Features of CD163+ Macrophage Infiltration

Meanwhile, univariate analyses of the predictors of pathological response demonstrated that gender and CD163+ macrophage infiltration were statistically significant in the validation cohort (Table 2). None of other clinical variables reached statistical significance. Meanwhile, variance inflation factor (VIF) analysis confirmed that no severe multicollinearity existed among the clinical variables included in the study (Table S5). Subsequently, multivariate regression analysis revealed that macrophage infiltration level was an independent predictor of NACT responses. Higher macrophage infiltration levels were significantly associated with a poorer NACT response.

Considering that our sample size was not sufficiently large, we further employed Firth regression analysis to reduce bias in small samples. The results showed that CD163+ macrophages, whether analyzed as a continuous variable or dichotomized by the median, were closely associated with a poor response to neoadjuvant chemotherapy in osteosarcoma and served as independent predictors of the efficacy of NACT (Tables S6 and S7).

**Figure 3:**
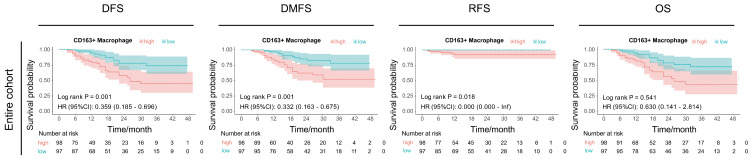
The performance of CD163+ macrophage infiltration in predicting survival in OS with NACT. The Kaplan–Meier survival curves for DFS, DMFS, RFS, and OS in the entire cohort.

We further investigated the association between macrophage infiltration and long-term survival outcomes. Patients were stratified into high- and low-infiltration groups based on median macrophage infiltration levels, with subsequent comparison of OS, DMFS, DFS, and RFS between groups. Notably, patients with higher CD163+ macrophage infiltration consistently demonstrated poorer survival trends across both training and validation cohorts, especially for DFS and DMFS (Figure S3A,B). When analyzing the entire cohort (*n* = 195), low CD163+ macrophage infiltration was significantly associated with better DFS (HR 0.359 95% CI: 0.185–0.696, *p* = 0.001), DMFS (HR 0.332 95% CI 0.163–0.675, *p* = 0.001), and RFS (HR < 0.001 95% CI 0.000–Inf, *p* = 0.018) (Figure 3). However, no statistically significant difference was observed in OS, which may be attributed to the relatively short follow-up duration.

### 3.4. Tumor Immune Microenvironment Profiles Across Differential Macrophage Infiltration Patterns

**Figure 4:**
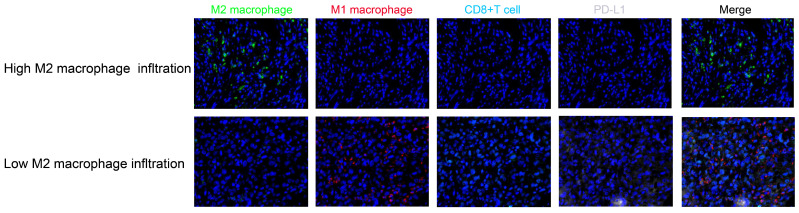
The different immunophenotypes in high and low CD163+ macrophage infiltration in OS. The presentative multiple immunofluorescence images of patients with high and low CD163+ macrophage infiltration.

To preliminarily investigate the tumor immune microenvironment in patients with different levels of CD163+ macrophage infiltration, we selected one representative patient from each group to perform multiplex immunofluorescence staining. Patients with high CD163+ macrophage infiltration demonstrated a more immunosuppressive microenvironment, characterized by significantly fewer T-cell infiltrates and lower PD-L1 expression than in the low-infiltration group (Figure 4). Collectively, these findings suggest that high CD163+ macrophage infiltration may be associated with an immune-exclusion tumor phenotype.

## 4. Discussion

Currently, neoadjuvant chemotherapy serves as a critical component of the standard treatment regimen for OS, significantly improving clinical survival outcomes [23,24]. However, the therapeutic efficacy of neoadjuvant chemotherapy exhibits considerable heterogeneity [25]. Moreover, no reliable predictive biomarkers are currently available to anticipate treatment response in patients with OS before surgical operation. The TIME plays a pivotal role in determining chemotherapy efficacy, and emerging evidence suggests that immunosuppressive macrophages are key regulators of chemotherapeutic response in OS [26,27]. Existing studies have demonstrated that macrophages play a critical role in the initiation, progression, therapeutic response, and distant metastasis of osteosarcoma [18,28,29]. Immunosuppressive macrophages, in particular, act in concert with tumor cells through reciprocal crosstalk, thereby influencing tumor metabolism and autophagy, which in turn exacerbates immune evasion and chemoresistance [30,31]. The functional status and infiltration levels of distinct macrophage subsets may play an important role in determining therapeutic response.

Preliminary evidence suggests that the enrichment of CD163+ macrophages (a primary marker of the M2 immunosuppressive phenotype) in pretreatment biopsies may actively drive a suboptimal response to NACT through multiple overlapping mechanisms [32,33]. Firstly, these macrophages secrete abundant anti-apoptotic factors and immunosuppressive cytokines (such as IL-10 and TGF-β), which can protect osteosarcoma cells from chemotherapy-induced cytotoxicity [33,34]. Secondly, CD163+ macrophages are known to stimulate pathological angiogenesis and induce extensive extracellular matrix (ECM) remodeling [35]. This remodeling can create a dense physical and interstitial fluid pressure barrier that restricts the effective delivery and deep penetration of chemotherapeutic agents into the tumor core. We hypothesize that the infiltration levels of immunosuppressive macrophages are closely associated with neoadjuvant chemotherapy outcomes. To test this hypothesis, we conducted a retrospective analysis using pretreatment biopsy samples from 195 patients with OS, investigating the relationship between immunosuppressive macrophage infiltration and NACT response.

To our knowledge, this represents a comprehensive study investigating the predictive value of immunosuppressive macrophage infiltration for NACT response in OS based on the pretreatment tumor tissues. Our findings suggest that pretreatment tumor macrophage infiltration may serve as a potential predictor of chemotherapeutic response. Across both training and validation cohorts, we consistently observed that patients with higher CD163+ macrophage infiltration demonstrated significantly reduced benefit from neoadjuvant chemotherapy (closely associated with non-MPR), while also having worse clinical outcomes than patients with low infiltration. Patients with increased CD163+ macrophage infiltration may consider alternative treatment options or actively participate in the latest clinical trials.

Furthermore, through multiplex immunofluorescence analysis, we systematically characterized the TIME in patients with varying levels of immunosuppressive macrophage infiltration. Our results demonstrated that high CD163+ macrophage infiltration was associated with a more immunosuppressive TIME, exhibiting significant depletion of CD8+ T cells, lower PD-L1 expression and M1-activated macrophage infiltration. These findings suggest that OS patients with predominant CD163+ macrophage infiltration display an “immune-exclusion” phenotype, whereas those with low infiltration maintain a more immunologically active microenvironment. This differential immune landscape implies that patients with low CD163+ macrophage infiltration may also potentially derive greater benefit from immunotherapy.

Previous studies have explored methods to effectively predict the response to NACT in OS, primarily focusing on imaging or serum biomarkers [36,37,38,39,40]. However, few have directly analyzed biopsy-derived tumor tissue samples. In contrast, our study is the first large-scale investigation to utilize TIME features for predicting NACT response, achieving robust predictive performance. Additionally, we conducted survival analyses, further validating the prognostic significance of CD163+ macrophage infiltration.

Nevertheless, our study has several limitations. First, the retrospective design and lack of external validation in an independent, multi-center cohort limit the immediate clinical applicability of the findings. Second, while we utilized pretreatment biopsies, these small tissue samples are highly susceptible to intra-tumoral spatial heterogeneity; therefore, a single core biopsy may not fully capture the complete and dynamic immune landscape of the entire tumor mass. Third, relying solely on a single immune cell infiltration metric may introduce analytical bias. A comprehensive evaluation of multiple immune cell populations and their spatial interactions would provide a more reliable predictive model. Fourth, our study is fundamentally observational. The exact molecular mechanisms by which CD163+ macrophages confer chemoresistance in OS require rigorous in vitro and in vivo functional verification. Finally, the event maturity in our cohort remains low, particularly for overall survival and recurrence. Extended, long-term follow-up is mandatory to definitively validate the prognostic impact of CD163+ macrophage infiltration.

Looking forward, future research should leverage advanced high-resolution technologies, such as single-cell RNA sequencing (scRNA-seq) and spatial transcriptomics, to deeply decode the phenotypic plasticity and precise spatial distribution of CD163+ macrophages within the OS microenvironment. Clinically, our findings pave the way for more-personalized therapeutic strategies. Integrating CD163+ macrophage infiltration scores with standard imaging and peripheral blood biomarkers could facilitate the development of a highly accurate, multiparametric predictive nomogram. Ultimately, exploring novel combinatorial therapies that specifically reprogram or deplete CD163+ macrophages alongside standard NACT hold significant promise for overcoming chemoresistance, reversing immune exclusion, and improving survival outcomes for patients with osteosarcoma.

## 5. Conclusions

Our study represents the first large-cohort investigation utilizing biopsy specimens to explore the association between CD163+ macrophage infiltration and NACT response in osteosarcoma, identifying it as an independent predictive biomarker for treatment efficacy. Furthermore, we comprehensively examined the relationships between CD163+ macrophage infiltration and various clinical characteristics, patient survival outcomes, and TIME features across different infiltration levels. These findings may provide valuable insights for precision therapy in osteosarcoma, suggesting that patients with varying degrees of CD163+ macrophage infiltration exhibit differential responses to NACT.

## Figures and Tables

**Table 1:** The clinicopathologic features of enrolled patients with OS.

Characteristics	Total (*n* = 195)	Training Cohort (*n* = 117)	Validation Cohort (*n* = 78)		*p*-Value
Age, *n* (%) years					0.778
<18	152 (77.95)	92 (78.63)	60 (76.92)		
≥18	43 (22.05)	25 (21.37)	18 (23.08)		
Mean ± SD	16.06 ± 8.70	15.98 ± 8.93	16.17 ± 8.40		0.879
Gender, *n* (%)					0.720
Male	118 (60.51)	72 (61.54)	46 (58.97)		
Female	77 (39.49)	45 (38.46)	32 (41.03)		
BMI, *n* (%)-kg/m^2^					0.152
Underweight or normal weight	131 (67.18)	74 (63.25)	57 (73.08)		
Overweight or obese	64 (32.82)	43 (36.75)	21 (26.92)		
Mean ± SD	20.37 ± 4.56	20.32 ± 4.10	20.46 ± 5.21		0.833
Location, *n* (%)					0.219
Right	97 (49.74)	54 (46.15)	43 (55.13)		
Left	98 (50.26)	63 (53.85)	35 (44.87)		
Limb, *n* (%)					0.926
Upper	22 (11.28)		13 (11.11)	9 (11.54)	
Lower	173 (88.72)		104 (88.89)	69 (88.46)	
Enneking stage, *n* (%)					1.000
IIB	180 (92.31)		108 (92.31)	72 (92.31)	
III	15 (7.69)		9 (7.69)	6 (7.69)	
Pathological subtype, *n* (%)					1.000
Conventional	188 (96.41)		113 (96.58)	75 (96.15)	
Others	7 (3.59)		4 (3.42)	3 (3.85)	
Pathological fracture, *n* (%)					0.639
Yes	182 (93.33)		110 (94.02)	72 (92.31)	
No	13 (6.67)		7 (5.98)	6 (7.69)	
Tumor length, *n* (%)-cm					0.559
<10	100 (51.28)		58 (49.57)	42 (53.85)	
≥10	95 (48.72)		59 (50.43)	36 (46.15)	
Mean ± SD	10.40 ± 4.35		10.26 ± 4.02	10.60 ± 4.81	0.598
Chemotherapy protocol, *n* (%)					0.166
MAPI	179 (91.79)		110 (94.02)	69 (88.46)	
MAPI + TKI	16 (8.21)		7 (5.98)	9 (11.54)	
Tumor necrosis rate, *n* (%)					0.769
<90%	105 (53.85)		64 (54.70)	41 (52.56)	
>90%	90 (46.15)		53 (45.30)	37 (47.44)	
Surgery type, *n* (%)					0.045
Limb salvage	183 (93.85)		106 (90.60)	77 (98.72)	
Amputation	12 (6.15)		11 (9.40)	1 (1.28)	
Recurrence, *n* (%)					1.000
Yes	190 (97.44)		114 (97.44)	76 (97.44)	
No	5 (2.56)		3 (2.56)	2 (2.56)	
Metastasis, *n* (%)					0.327
Yes	159 (81.54)		98 (83.76)	61 (78.21)	
No	36 (18.46)		19 (16.24)	17 (21.79)	
Survival *n* (%)					1.000
Alive	188 (96.41)		113 (96.58)	75 (96.15)	
Dead	7 (3.59)		4 (3.42)	3 (3.85)	

Note: SD—standard deviation.

**Table 2:** Univariate and multivariate analyses of various predictors for pathological response in training and validation cohorts.

Characteristics	Univariable Analysis				Multivariate Analysis			
	Training Cohort		Validation Cohort		Training Cohort		Validation Cohort	
	*p*	OR (95%CI)	*p*	OR (95%CI)	*p*	OR (95%CI)	*p*	OR (95%CI)
**CD163+ macrophage density/mm^2^**								
Low infiltration		1.00 (Reference)		1.00 (Reference)		1.00 (Reference)		1.00 (Reference)
High infiltration	<0.001	37.74 (12.95~109.97)	<0.001	15.24 (5.04~46.06)	<0.001	61.33 (16.58~226.85)	<0.001	29.00 (6.41~131.14)
**Location**								
Right		1.00 (Reference)		1.00 (Reference)		1.00 (Reference)		1.00 (Reference)
Left	0.36	0.71 (0.34~1.48)	0.856	0.92 (0.38~2.25)	0.599	1.39 (0.41~4.74)	0.816	0.85 (0.22~3.29)
**Limb**								
Upper		1.00 (Reference)		1.00 (Reference)		1.00 (Reference)		1.00 (Reference)
Lower	0.513	1.47 (0.46~4.68)	0.849	0.87 (0.22~3.53)	0.518	2.02 (0.24~17.10)	0.326	0.37 (0.05~2.70)
**Gender**								
Male		1.00 (Reference)		1.00 (Reference)		1.00 (Reference)		1.00 (Reference)
Female	0.597	1.22 (0.58~2.59)	0.028	0.35 (0.14~0.89)	0.848	1.12 (0.34~3.66)	0.080	0.29 (0.07~1.16)
**Pathological fracture**								
Yes		1.00 (Reference)		1.00 (Reference)		1.00 (Reference)		1.00 (Reference)
No	0.52	0.60 (0.13~2.82)	0.338	0.42 (0.07~2.46)	0.719	1.55 (0.14~16.65)	0.062	0.10 (0.01~1.12)
**Age**								
<18		1.00 (Reference)		1.00 (Reference)		1.00 (Reference)		1.00 (Reference)
≥18	0.883	1.07 (0.44~2.60)	0.190	0.49 (0.17~1.43)	0.673	0.73 (0.17~3.18)	0.214	0.36 (0.07~1.80)
**BMI (kg/m^2^)**								
Underweight or normal weight		1.00 (Reference)		1.00 (Reference)		1.00 (Reference)		1.00 (Reference)
Overweight or obese	0.854	1.07 (0.50~2.29)	0.318	1.68 (0.61~4.68)	0.655	1.30 (0.41~4.14)	0.360	0.46 (0.09~2.42)
**Tumor length (cm)**								
<10		1.00 (Reference)		1.00 (Reference)		1.00 (Reference)		1.00 (Reference)
≥10	0.399	0.73 (0.35~1.52)	0.624	1.25 (0.51~3.05)	0.057	0.29 (0.08~1.04)	0.963	0.97 (0.26~3.58)
**Chemotherapy protocol**								
MAPI		1.00 (Reference)		1.00 (Reference)		1.00 (Reference)		1.00 (Reference)
MAPI + TKI	0.369	2.16 (0.40~11.62)	0.605	0.69 (0.17~2.80)	0.26	4.02 (0.36~45.31)	0.758	1.40 (0.16~11.86)
**Pathological subtype**								
Conventional		1.00 (Reference)		1.00 (Reference)		1.00 (Reference)		1.00 (Reference)
Others	0.989	Inf	0.990	Inf	0.991	Inf	0.993	Inf
**Enneking stage**								
IIB		1.00 (Reference)		1.00 (Reference)		1.00 (Reference)		1.00 (Reference)
III	0.957	1.04 (0.26~4.08)	0.896	0.89 (0.17~4.73)	0.937	0.91 (0.09~8.99)	0.344	0.32 (0.03~3.40)

OR: odds ratio, CI: confidence interval.

## Data Availability

The datasets used and analyzed during the current study are available from the corresponding author on request.

## Supplementary Materials

The following supporting information can be downloaded at https://www.mdpi.com/article/10.3390/biomedicines14050991/s1: Figure S1. The flowchart of patient screening for final inclusion in this study. Figure S2. The Kaplan–Meier analyses of DFS, MDFS, RFS, and OS in the MPR and non-MPR groups in the entire (A), training (B), and validation (C) cohorts. Figure S3. The Kaplan–Meier analyses of DFS, MDFS, RFS, and OS in the MPR and non-MPR groups in the training (A) and validation (B) cohorts. Table S1. The details of full ROC metrics for CD163+ macrophage density (median-dichotomized) in the training and validation cohorts. Table S2. Univariate analysis of CD163+ macrophage density (continuous, median-dichotomized, log-transformed) in predicting TNR of osteosarcoma. Table S3. The details of CD163+ macrophage density (continuous and median-dichotomized) of calibration slope and Brier score in predicting TNR of osteosarcoma. Table S4. The details of CD163+ macrophage density (continuous and median-dichotomized) of calibration slope and Brier score in predicting TNR of osteosarcoma excluding patients receiving MAPI + TKI therapy. Table S5. The details of variance inflation assessments for enrolled clinical variables in this study. Table S6. Firth penalized regression of CD163+ macrophage density (median-dichotomized) in predicting TNR of osteosarcoma. Table S7. Firth penalized regression of CD163+ macrophage density (per 100 cells/mm^2^) in predicting TNR of osteosarcoma.

## Abbreviations

OS	Osteosarcoma
MPR	Major pathological response
NACT	Neoadjuvant chemotherapy
TIME	Tumor immune microenvironment
TAMs	Tumor-associated macrophages
MDSCs	Myeloid-derived suppressor cells
IHC	Immunohistochemistry
mIF	Multiplex immunofluorescence
RFS	Recurrence-free survival
DFS	Disease-free survival
DMFS	Distant metastasis-free survival
FFPE	Formalin-fixed, paraffin-embedded
