# Supplementary material for: Prognostic and Predictive Value of CD163+ Macrophages for Neoadjuvant Chemotherapy in Osteosarcoma
Source: Biomedicines. 2026 Apr 26;14(5):991. doi: 10.3390/biomedicines14050991 (PMC13204522; doi:10.3390/biomedicines14050991)
Supplement: Supplementary file 1 [file biomedicines-14-00991-s001.zip › biomedicines-4182392-supplementary.pdf]

Figure S1. The flowchart of patient screening for final inclusion in this study.

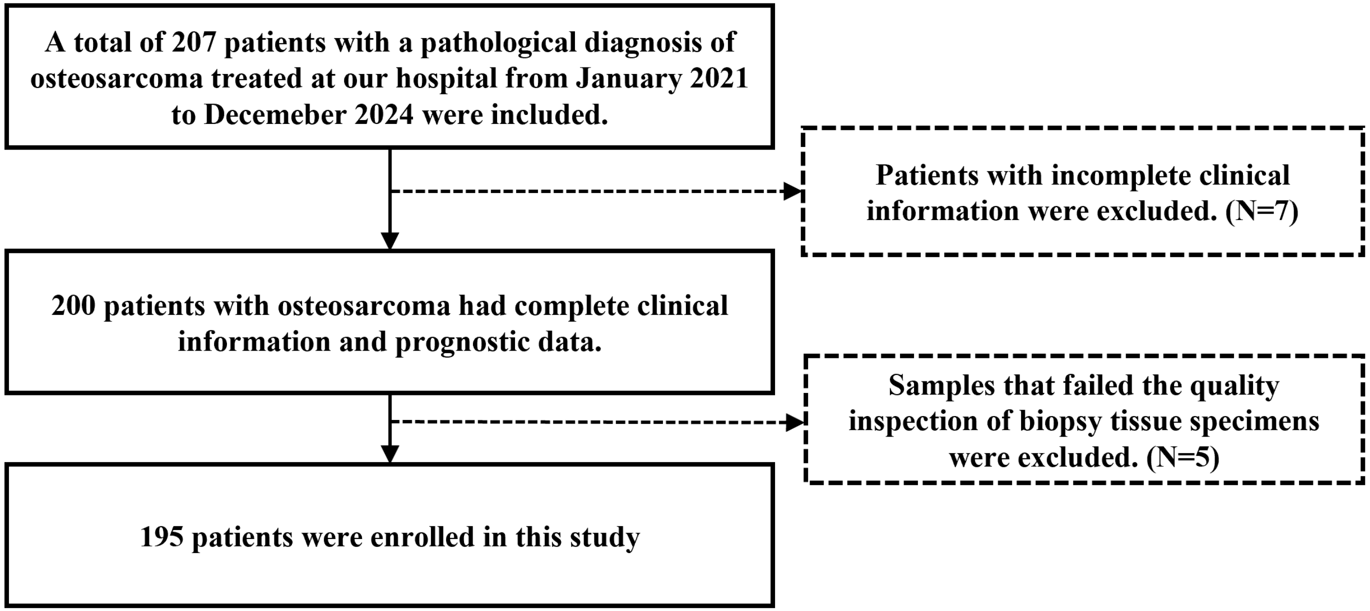

Figure S2. The Kaplan-Meier analyses of DFS, MDFS, RFS, and OS in the MPR and non-MPR groups in the entire (A), training (B), and validation (C) cohorts.

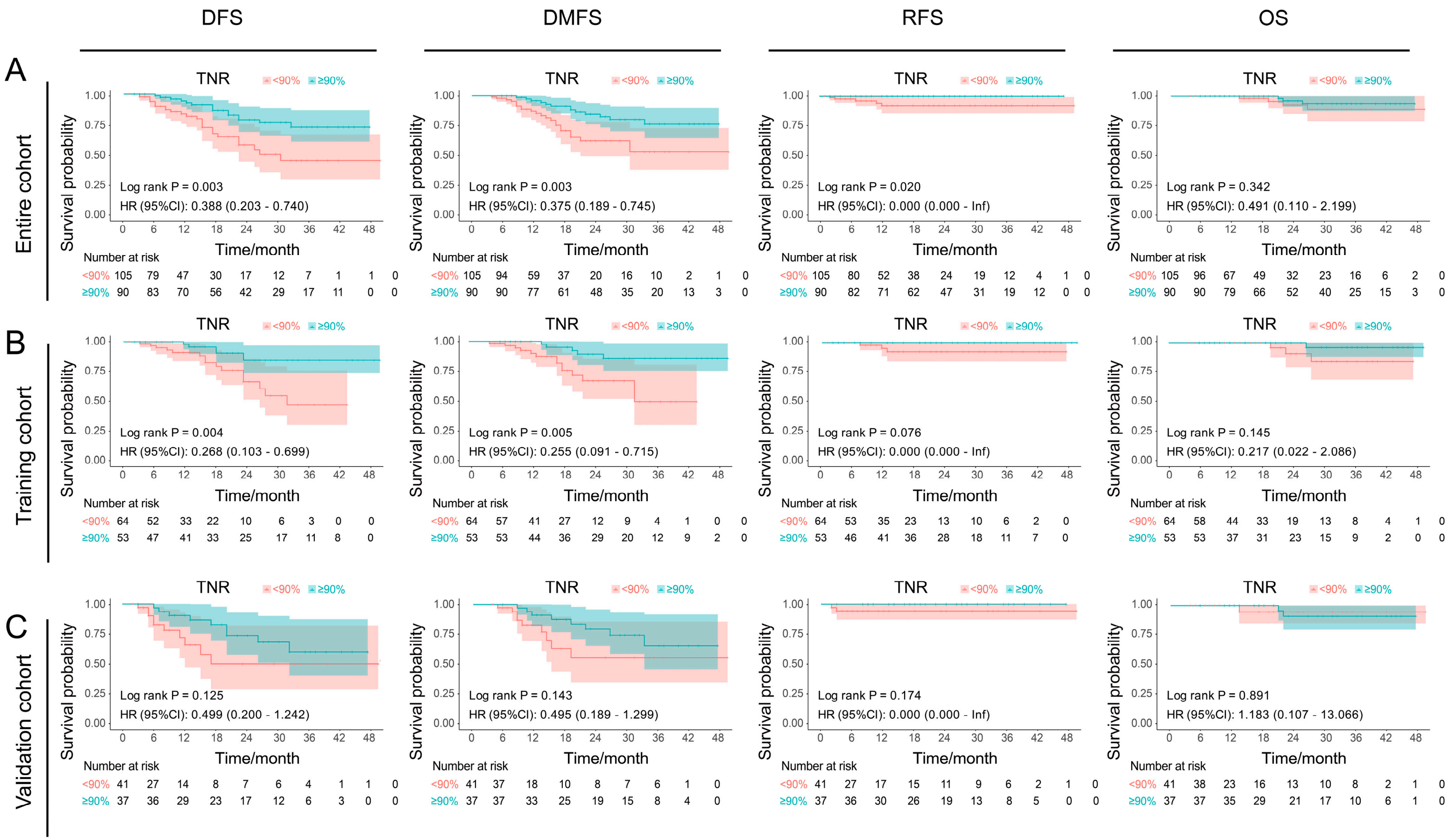

Figure S3. The Kaplan-Meier analyses of DFS, MDFS, RFS, and OS in the MPR and non-MPR groups in the training (A) and validation (B) cohorts.

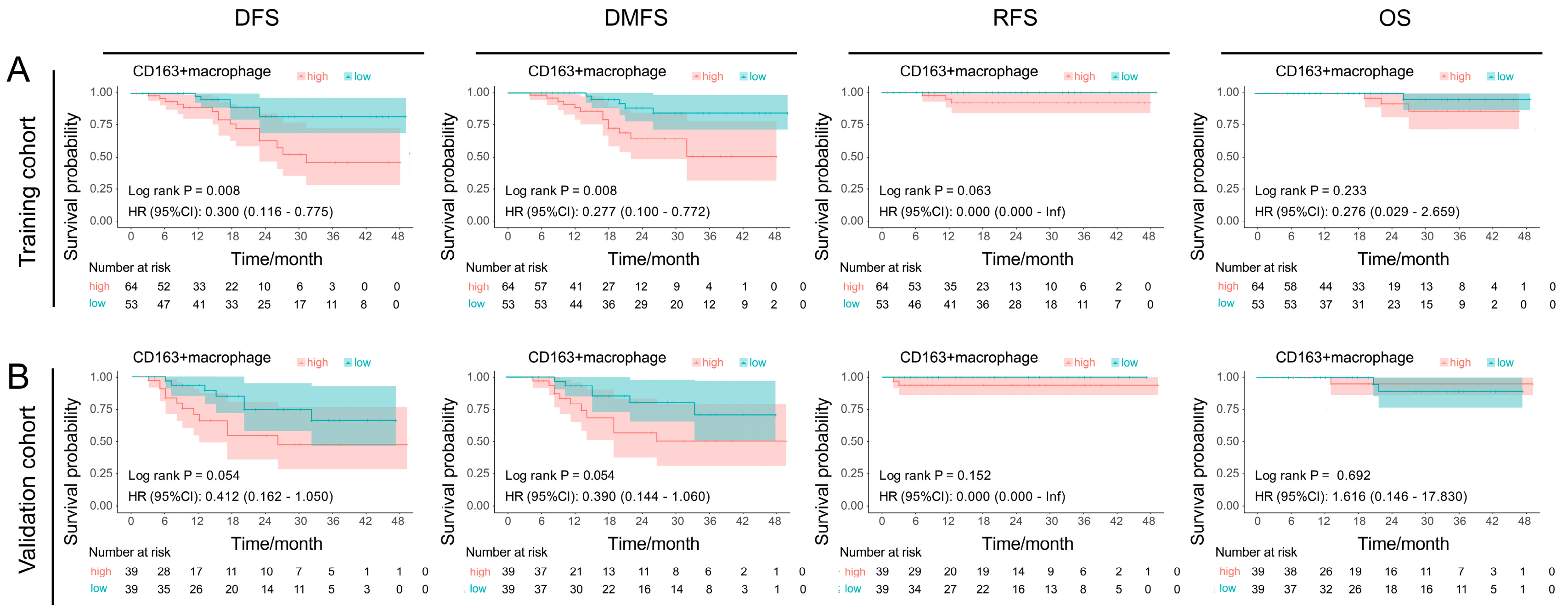

Table S1. The details of full ROC metrics for CD163⁺macrophage density (median-dichotomized) in the training and validation cohorts

Cohort	AUC (95%CI)	Accuracy (95%CI)	Sensitivity (95%CI)	Specificity (95%CI)	PPV (95%CI)	NPV (95%CI)	Optimal Youden index
Training cohort	0.92 (0.87-0.97)	0.86 (0.79-0.92)	0.89 (0.80-0.97)	0.84 (0.75-0.93)	0.82 (0.73-0.92)	0.90 (0.82-0.98)	0.678
Validation cohort	0.82 (0.72-0.92)	0.81 (0.70-0.89)	0.81 (0.68-0.94)	0.80 (0.68-0.93)	0.79 (0.66-0.92)	0.82 (0.71-0.94)	0.678

Table S2. Univariate analysis of CD163+ macrophage density (continuous, median-dichotomized, log-transformed) in predicting TNR of Osteosarcoma

Characteristics	Training cohort		Validation cohort	
	P value	OR	P value	OR
CD163+ macrophage density (per 100 cells/mm^2)	<0.001	1.374(1.214-1.616)	<0.001	1.123(1.046-1.237)
Log(1+CD163+ macrophage density)	<0.001	4.053(2.586-7.128)	<0.001	2.351(1.575-3.863)

Table S3. The details of for CD163$^+$macrophage density (continuous and median-dichotomized) of calibration slope and Brier score in predicting TNR of Osteosarcoma

Characteristics	Training cohort		Validation cohort	
	Calibration slope	Brier score	Calibration slope	Brier score
CD163+macrophage density (per 100 cells/mm^2)	1.249	0.124	0.401	0.212
CD163+macrophage density (High/Low infiltration)	1.185	0.112	0.644	0.197

Table S4. The details of for CD163$^+$macrophage density (continuous and median-dichotomized) of calibration slope and Brier score in predicting TNR of Osteosarcoma excluding patients receiving MAPI+TKI therapy.

Characteristics	Training cohort		Validation cohort	
	Calibration slope	Brier score	Calibration slope	Brier score
CD163+macrophage density (per 100 cells/mm^2)	1.230	0.124	1.270	0.192
CD163+macrophage density (High/Low infiltration)	1.185	0.112	0.644	0.197

Table S5. The details of variance inflation assessments for enrolled clinical variables in this study.

Characteristics	VIF
CD163+macrophage density (High/Low infiltration)	1.056
Location	1.123
Limb	1.166
Gender	1.100
Pathological fracture	1.058
BMI (kg/m^2)	1.094
Tumor length (cm)	1.044
Chemotherapy protocol	1.039
Pathological subtype	1.12
Enneking stage	1.033

Table S6. Firth penalized regression of CD163+ macrophage density (median-dichotomized) in predicting TNR of Osteosarcoma

Characteristics	Training cohort		Validation cohort	
	P value	OR	P value	OR
**CD163+ macrophage density**				
High infiltration				
Low infiltration	<0.001	32.840(11.659-115.118)	<0.001	7.758(2.552-29.048)
**Location**				
Right				
Left	0.673	0.787(0.243-2.368)	0.597	1.335(0.459-4.050)
**Limb**				
Upper				
Lower	0.547	1.846(0.263-2.368)	0.484	0.567(0.110-2.862)
**Gender**				
Male				
Female	0.824	0.883(0.291-2.679)	0.127	2.439(0.777-8.040)
**Pathological fracture**				
Yes				
No	0.729	1.472(0.138-11.655)	0.363	0.429(0.051-2.599)
**Age**				
＜18				

≥18	0.674	0.747(0.180-2.877)	0.473	0.623(0.154-2.242)
**BMI (kg/m²)**				
Underweight or Normal weight				
Overweight or Obese	0.662	1.267(0.435-3.732)	0.473	0.593(0.136-2.270)
**Tumor length (cm)**				
＜10				
≥10	0.070	0.366(0.105-1.083)	0.609	1.322(0.450-3.916)
**Chemotherapy protocol**				
MAPI				
MAPI+TKI	0.351	2.874(0.317-29.496)	0.850	1.164(0.233-5.837)
**Pathological subtype**				
Conventional				
Others	0.069	23.034(0.790-4126.195)	0.327	4.549(0.285-816.383)
**Enneking stage**				
IIB				
III	0.940	0.920(0.111-7.665)	0.316	0.366(0.046-2.685)

Table S7. Firth penalized regression of CD163+ macrophage density (per 100 cells/mm²) in predicting TNR of Osteosarcoma

Characteristics	Training cohort		Validation cohort	
	P value	OR	P value	OR
**CD163+ macrophage density (per 100 cells/mm²)**	<0.001	1.332(1.189-1.567)	0.007	1.102(1.025-1.215)
**Location**				
Right				
Left	0.776	0.864(0.307-2.345)	0.585	1.322(0.486-3.700)
**Limb**				
Upper				
Lower	0.251	3.003(0.482-29.856)	0.332	0.475(0.098-2.170)
**Gender**				
Male				
Female	0.526	0.725(0.266-1.980)	0.132	2.297(0.780-7.113)
**Pathological fracture**				
Yes				
No	0.918	0.901(0.096-6.134)	0.646	0.681(0.109-3.429)
**Age**				
<18				
≥18	0.851	0.889(0.248-2.986)	0.306	0.534(0.145-1.754)

**BMI (kg/m²)**				
Underweight or Normal weight				
Overweight or Obese	0.636	1.261(0.479-3.311)	0.672	0.762(0.208-2.683)
**Tumor length (cm)**				
＜10				
≥10	0.136	0.482(0.172-1.251)	0.905	1.064(0.381-2.968)
**Chemotherapy protocol**				
MAPI				
MAPI+TKI	0.562	1.918(0.195-17.932)	0.959	0.962(0.213-4.328)
**Pathological subtype**				
Conventional				
Others	0.116	9.541(0.609-1500.808)	0.148	6.344(0.567-869.115)
**Enneking stage**				
IIB				
III	0.557	1.642(0.299-8.783)	0.572	0.584(0.075-3.816)
